# 288. Analysis of serum antibody response as correlates of protection against SARS-CoV-2 infection and the effects of BA.4/5 bivalent booster vaccination

**DOI:** 10.1093/ofid/ofad500.360

**Published:** 2023-11-27

**Authors:** So Yun Lim, Jineui Kim, Ji-Soo Kwon, Sung-Woon Kang, Eui Jin Chang, Seongman Bae, Min Jae Kim, Yong Pil Chong, Sang-Oh Lee, Sang-Ho Choi, Man-Seong Park, Sung-Han Kim

**Affiliations:** National Medical Center , Seoul, Seoul-t'ukpyolsi, Republic of Korea; Korea university, Seoul, Seoul-t'ukpyolsi, Republic of Korea; Department of Infectious Disease, Asan Medical Center, University of Ulsan College of Medicine, Seoul, Seoul-t'ukpyolsi, Republic of Korea; Asan Medical Center, Seoul, Seoul-t'ukpyolsi, Republic of Korea; Department of Internal Medicine, Asan Medical Center, Seoul, Korea, Seoul, Seoul-t'ukpyolsi, Republic of Korea; Asan Meidical Center, Songpa-gu, Seoul-t'ukpyolsi, Republic of Korea; Asan Medical Center, Seoul, Seoul-t'ukpyolsi, Republic of Korea; Asan Medical Center, Seoul, Seoul-t'ukpyolsi, Republic of Korea; Asan Medical Center, Seoul, Seoul-t'ukpyolsi, Republic of Korea; Asan Medical Center, Seoul, Seoul-t'ukpyolsi, Republic of Korea; Korea University, Seoul, Seoul-t'ukpyolsi, Republic of Korea; Asan medical center, Seoul, Seoul-t'ukpyolsi, Republic of Korea

## Abstract

**Background:**

Serum anti-spike IgG and neutralizing antibody against SARS-CoV-2 are recognized as immune correlates of protection (ICP) for COVID-19. However, there are limited data available on the ICP after the introduction of BA.4/5 bivalent vaccine. Thus, our study aimed to investigate the antibody response as correlates of protection against COVID-19, and the effects of BA.4/5 bivalent booster vaccine.

**Methods:**

A prospective cohort of healthcare workers was enrolled at Asan Medical Center, a 2,700-bed tertiary care hospital in South Korea between December 2022 and January 2023. Study participants had received a booster vaccination and either planned to receive a bivalent BA.4/5 vaccine or not. Blood samples were collected from study participants. Immunological assessments were performed using ELISA to measure variant-specific SARS-CoV-2 S1-IgG antibodies, and virus reduction neutralization test (VRNT) to measure neutralizing antibody levels.

**Figure 1.**
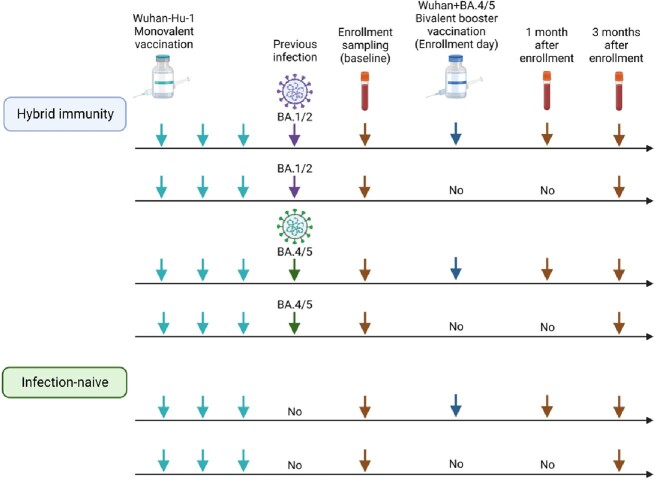
Study design

**Results:**

A total of 483 participants were enrolled, of whom 45 (9.3%) underwent subsequent infection after study enroll, while 438 (90.7%) did not. Of these 483 participants, 166 (34.4%) received the BA.4/5 bivalent booster vaccination. There was a significant difference in Wuhan-Hu-1 S1-IgG, BA.5 S1-IgG and neutralizing antibody against BA.5 levels between individuals with and without subsequent infection (all P < 0.001). While Wuhan S1-IgG and BA.5 S1-IgG (both P < 0.001) were found to be independent protective factors against subsequent SARS-CoV-2 infection, neutralizing antibody against BA.5 did not show significant protective effects. Hybrid immunity was robust predictive factor for subsequent infection in all analysis based on each immune marker (all P < 0.001). The predictive sensitivity of neutralizing antibody for subsequent infection was improved from 80.0% (95% CI, 60.0-95.0) to 90.0% (95% CI, 68.3-98.8) when combined with hybrid immunity and 100% (95% CI, 83.2-100) with bivalent vaccine.

**Table 1.**
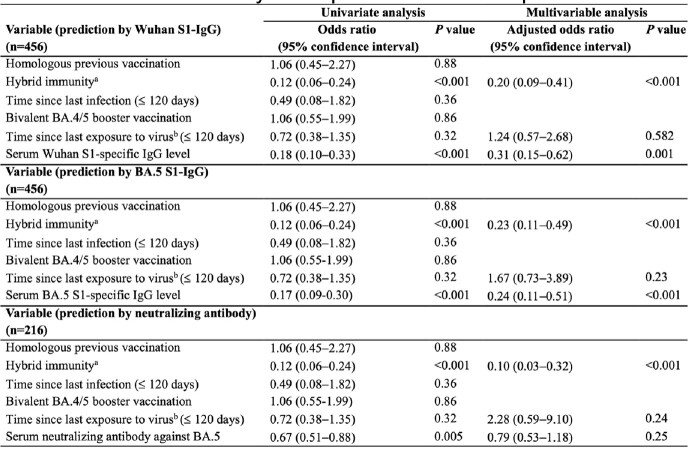
Multivariable analysis for prediction of subsequent infection after study enrollment. aCompared to participants with infection-naïve bEither vaccination or infection

**Table 2.**
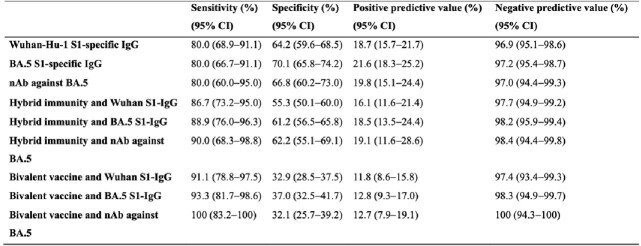
Predictive performance of each immune markers at optimal cut-off value for SARS-CoV-2 subsequent infection. Abbreviation: nAb, neutralizing antibody

**Figure 2.**
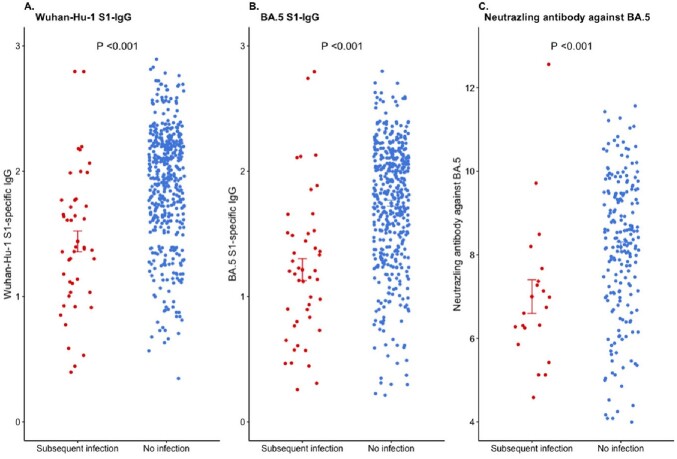
Comparison of baseline antibody titer according to the subsequent SARS-CoV-2 infection. A. Wuhan-Hu-1 S1-specific IgG B. BA.5 S1-specific IgG C. Neutralizing antibody against BA.5

**Conclusion:**

Taken together, humoral immune responses seem to be reliable markers for immune correlates of protection against SARS-CoV-2 infection, and hybrid immunity showed independent protective effect from subsequent infection.

**Figure 3.**
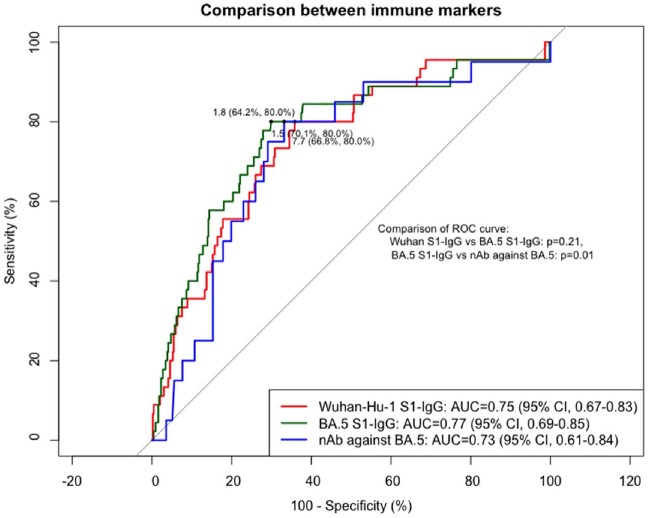
Receiver operating curves of Wuhan-Hu-1 S1-IgG, BA.5 S1-IgG, and neutralizing antibody against BA.5 in predicting subsequent infection. Black dots indicate optimal cut-off value by Youden index method and the two numbers in parentheses refer to specificity and sensitivity, sequentially.

**Disclosures:**

**All Authors**: No reported disclosures

